# Should we remove flexible intramedullary nails after diaphyseal forearm pediatric fracture? A cohort study of 288 patients

**DOI:** 10.2340/17453674.2026.46804

**Published:** 2026-09-14

**Authors:** Maria TIRTA, Amalie EIKREM-HOLSÆTER, Eline Borg HOLT, Richard OLSSON, Rune Bruhn JAKOBSEN, Per-Henrik RANDSBORG

**Affiliations:** 1 Department of Orthopedic Surgery, Akershus University Hospital, Oslo, Norway; 2 Interdisciplinary Orthopaedics, Aalborg University Hospital, Aalborg, Denmark; 3 Faculty of Medicine, University of Oslo, Oslo, Norway

## Abstract

**Background and purpose:**

Routine removal of elastic stable intramedullary nails (ESIN) after pediatric diaphyseal forearm fractures remains common practice despite limited supporting evidence. We aimed to evaluate refracture risk following ESIN retention compared with implant removal and assessed complications and healthcare resource use.

**Methods:**

In this retrospective cohort study, patients treated with ESIN for diaphyseal forearm fractures at a single center were included. Following a policy change, routine implant removal was discontinued in favor of retaining implants unless symptomatic. Patients were grouped into removal and non-removal. The primary outcome was refracture, analyzed using Cox regression, and early refractures (< 12 months), using logistic regression. Secondary outcomes included complications and healthcare resource utilization (outpatient visits, radiographic examinations during follow-up).

**Results:**

288 patients were included, of whom 136 underwent implant removal and 152 retained their implants. Among patients with implant retention, 9/152 (5.9%) subsequently required removal, most commonly due to implant-related irritation. Refracture occurred in 13/136 (9.6%) after implant removal and 8/152 (5.3%) after implant retention (absolute risk difference 4.3 percentage points, 95% confidence interval [CI] −1.8 to 10.4; HR 1.4, CI 0.5–3.4). Early refracture occurred in 8/136 (5.9%) patients after implant removal and 5/152 (3.3%) after implant retention (absolute risk difference 2.6 percentage points, CI −2.3 to 7.5; OR 1.8, CI 0.6–6.2), including 3 cases within 6 months after removal. Complications occurred in 11/136 (8.1%) patients after implant removal. Infection occurred in 5/136 (3.7%) after implant removal vs 4/288 (1.4%) after the initial surgery (risk difference 2.3 percentage points, CI −1.2 to 5.7).

**Conclusion:**

ESIN retention was well tolerated and associated with reduced healthcare utilization. No clear difference in refracture risk was observed between groups, although the study was not powered to detect moderate differences in risk.

Forearm diaphyseal fractures are among the most common injuries in children, accounting for approximately 3–5.4% of all pediatric fractures [1,2]. While most forearm fractures are treated conservatively with satisfactory outcomes, diaphyseal fractures are often associated with higher instability and greater likelihood of requiring surgical treatment [3,4].

The primary surgical method is elastic stable intramedullary nailing (ESIN), a well-established, minimally invasive technique that provides elastic stable fixation while preserving soft tissues and periosteal blood supply [5]. It is widely used in the treatment of pediatric diaphyseal forearm fractures and has demonstrated favorable clinical outcomes [5,6].

Until recently, the standard practice has been routine second surgery for implant removal once the fracture is sufficiently healed, suggested after 6 months [6,7]. However, there is limited evidence to support whether routine removal of ESIN is necessary in pediatric forearm fractures [8]. A recent study reported high rates of long-term implant retention (83%), as implants were not routinely removed, with secondary procedures required in 19% of patients, most commonly due to local irritation prompting implant removal [8].

Implant removal is not without risk. It constitutes an additional surgery, exposing patients to perioperative risks such as neurovascular complications and infections, with a reported complication rate of around 3% [9,10]. In addition, it necessitates further healthcare utilization, including additional outpatient visits and radiographic examinations, resulting in increased radiation exposure.

The above-mentioned factors call into question the necessity of routine implant removal, particularly given that implant removal is not routinely performed after internal fixation of fractures in adults. Accordingly, the primary aim of this study was to evaluate whether implant retention is associated with similar risk of refracture compared with routine implant removal. Secondary outcomes included the number of radiographic examinations and overall follow-up resource utilization.

## Methods

### Study design and setting

This study was designed as a single-center retrospective cohort study. The STROBE guidelines for cohort studies were followed in the reporting of this study [11]. The study was conducted at Akershus University Hospital, a tertiary referral center. All patients treated with elastic stable intramedullary nailing (ESIN) for forearm shaft fractures during the years 2017, 2018, and 2022–2024 were eligible for inclusion. The period 2019–2021 was excluded due to the impact of the COVID-19 pandemic on surgical activity and treatment patterns.

ESIN was performed according to established principles of elastic stable intramedullary nailing as described in the AO Surgery Reference [12]. Nails were inserted retrograde in the radius and antegrade in the ulna. Closed reduction was attempted in all cases; however, mini-open reduction was performed when satisfactory reduction could not be achieved by closed means.

A change in institutional policy occurred after 2021, whereby routine removal of ESIN implants was discontinued. Consequently, the nail ends were cut shorter and left flush with the cortex to minimize soft tissue irritation when implants were intended to remain in situ. Implant removal was thereafter performed only in symptomatic patients defined as patients with implant-related pain, soft tissue irritation, implant prominence, or other implant-related symptoms considered to warrant removal. No other changes were made to the surgical technique or indications for surgery. Procedures were performed by multiple surgeons at this tertiary teaching hospital under the supervision of 2 pediatric orthopedic consultants, who remained responsible for the management of these fractures throughout the study period.

Patients were identified using the Nordic Medico-Statistical Committee (NOMESCO) Classification of Surgical Procedures (NCSP) codes, corresponding to osteosynthesis of a forearm fracture with intramedullary nail or pin: NCJ4 (and all subcodes) and NCJ5 (and all subcodes). All data was anonymized prior to analysis. The end of follow-up for all patients was defined as January 31, 2026.

### Participants

All pediatric patients younger than 17 years, treated with flexible intramedullary nails for primary forearm shaft fractures during the study period, were screened for eligibility. Patients were included if they had undergone ESIN fixation for a primary diaphyseal forearm fracture and had available follow-up data.

Patients were excluded if they had (i) other surgical treatment (e.g., pin fixation), (ii) primary treatment performed at another hospital or were visiting patients, (iii) fracture types not relevant to the study (refractures without a primary fracture during the study period, Monteggia or Galeazzi fractures, radial head or pathological fractures), or (iv) insufficient clinical records or incomplete radiographic follow-up.

### Follow-up protocol

Patients were followed up according to standard institutional practice, with outpatient clinical and radiographic assessments after 6 weeks and, if needed, until radiographic fracture healing. Before 2021, patients were routinely scheduled for nail removal approximately 6 months after the index surgery. Additional follow-up visits were scheduled as clinically indicated, including for assessment of complications or implant-related symptoms. Information on follow-up duration and healthcare utilization was collected from medical records. Follow-up for refracture commenced at the date of the index ESIN surgery and continued until refracture or the end of available follow-up. Patient identification was performed using standardized procedure codes. Radiographic records were reviewed to confirm refracture events. All available follow-up data was included to reduce the risk of outcome misclassification. All eligible patients treated during the study period were included in the analysis.

### Variables

The primary grouping variable was actual implant management during follow-up. Patients were classified in the removal group if the implants were removed during follow-up and in the retention group if the implants remained in situ. Implant removal was performed after fracture healing and occurred at variable time points during follow-up.

Additional variables collected were age, sex, height, weight, and body mass index (BMI). Fracture-related variables included fracture type (both-bone forearm fracture, isolated radial shaft fracture, isolated ulnar shaft fracture), presence of open fracture (yes/no), and associated injuries (yes/no). Clinical variables included preoperative nerve injury (yes/no), operative time (minutes), and reoperation (yes/no). Detailed data on refracture events was collected, including time to refracture (months from primary surgery), time from implant removal to refracture (when applicable), and management of the refracture (e.g., casting, reoperation with ESIN, or plate fixation).

### Outcomes

The primary outcome was refracture of the forearm. Refractures were further categorized according to time from the primary surgery into early (< 12 months) and late (≥ 12 months) events, based on the hypothesis that refracture risk may differ over time following fracture healing.

Secondary outcomes included postoperative complications and healthcare resource use. Complications were recorded as infection, nerve injury, malposition of the ESIN, malreduction, bleeding, reoperation, refracture within 3 months, and other complications. Healthcare resource use was defined as the number of outpatient visits (0, 1, 2, ≥ 3 visits) and the number of radiographic examinations (≤ 3, 4–5, ≥ 6) following the primary surgery.

### Data sources

Clinical variables were extracted from electronic medical records. Radiographic data was obtained from the hospital imaging system and reviewed to confirm fracture type and identify refracture events.

Data was recorded on a secure digital research platform (Ledidi AS, Oslo, Norway), a secure digital platform for structured clinical data collection and management, hosted within the institution’s protected IT infrastructure.

### Statistics

Descriptive statistics were used to summarize the study population. Continuous variables are presented as mean with standard deviation (SD) for normally distributed data or median with interquartile range (IQR) for non-normally distributed data. Categorical variables are presented as counts and percentages. The distribution of continuous variables was assessed for normality using the Shapiro–Wilk test and visual inspection of histograms and Q–Q plots. No formal sample size calculation was performed due to the retrospective design.

The primary outcome, overall refracture during follow-up, was analyzed using Cox proportional hazards regression to account for differences in follow-up time. Follow-up time was calculated from the index ESIN surgery to refracture or censoring, and patients were analyzed according to their actual implant management group as defined above. Patients who subsequently underwent implant removal were classified in the removal group for the analysis, irrespective of the timing of removal during follow-up. Results were reported as hazard ratios (HR) with 95% confidence intervals (CI). Group-specific risks and absolute risk differences (RD) with 95% CI, calculated using the Wald method, were also reported to provide absolute measures of between-group differences. Refracture-free survival over time was illustrated using Kaplan–Meier curves.

As secondary analyses, early refracture occurring within 12 months and late refracture (≥ 12 months) were evaluated in separate univariable logistic regression models, with results reported as odds ratios (OR) with 95% CI. Absolute risk differences with 95% CI were also calculated for these outcomes.

Due to the limited number of events, analyses were restricted to univariable models. Differences in follow-up resource use, including the number of outpatient visits and radiographic examinations, were analyzed as categorical variables and compared between groups using the chi-square test. Absolute risk differences with 95% CI were additionally reported for selected clinically relevant resource-use outcomes. The difference in infection rates following the initial ESIN procedure and implant removal was analyzed using Fisher’s exact test.

Missing data was assessed and reported in accordance with current statistical reporting recommendations [13]. There was no missing data for the primary or principal secondary outcomes. Missing data was limited to selected baseline and perioperative variables: BMI was unavailable for 29 patients because height was not recorded (13 in the retention group and 16 in the removal group), and operative time was missing for 6 patients. Available-case analysis was used for variables with missing observations, and no imputation was performed. Because outcome data was complete, no sensitivity analysis for missing outcome data was performed.

All statistical tests were 2-sided, and a P value < 0.05 was considered statistically significant. Statistical analyses were performed using R (R Foundation for Statistical Computing, Vienna, Austria).

### Ethics, data sharing, funding, AI use, and disclosures

The study was approved by the regional ethics committee (961151) and local data protection officer (#2025-174). Due to the retrospective design, the requirement for informed consent was waived. The data underlying this study is available from the corresponding author upon reasonable request and subject to institutional data protection regulations. This research received no external funding. The authors declare no conflict of interest. During the preparation of this manuscript, the authors used an artificial intelligence–based language tool to improve clarity and readability of the text, but it was not used in the analysis of data. The authors reviewed and edited the output and take full responsibility for the content of the manuscript. Complete disclosure of interest forms according to ICMJE are available on the article page, doi: 10.2340/17453674.2026.46804

## Results

### Participants and baseline characteristics

**Figure 1:**
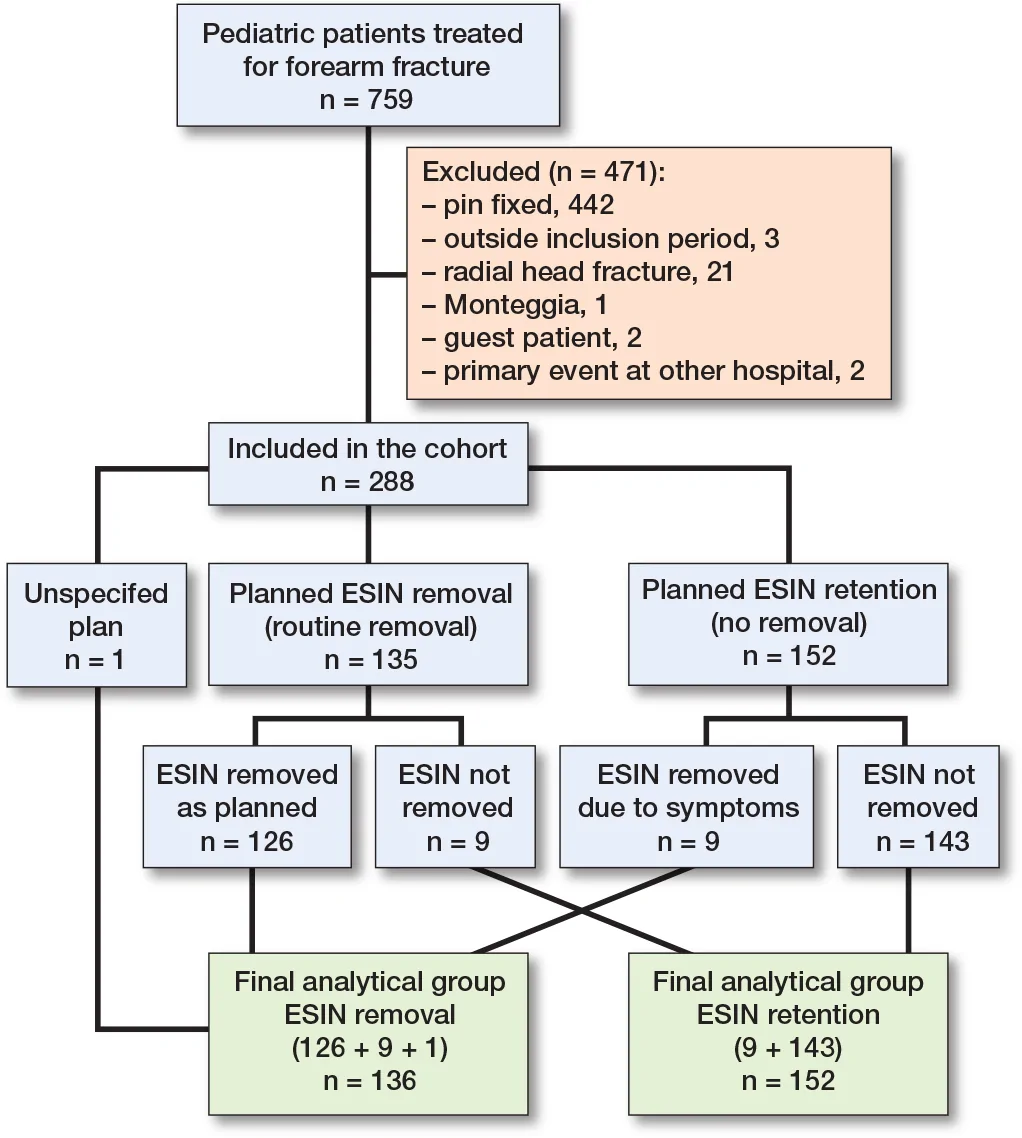
Flowchart of patient inclusion and exclusion in the study cohort.

Of the 759 potential patient records, a total of 288 patients treated with flexible intramedullary nails (ESIN) for forearm fractures were identified, of whom 136 (47%) underwent subsequent implant removal and 152 (53%) retained their implants (Figure 1).

Baseline characteristics are presented in Table 1. The mean age was 9.2 (SD 3.0) years, 59% were male, and most fractures involved both forearm bones (84%). Baseline characteristics were similar between groups (Table 1). The most common injury mechanisms were falls (26%) and trampoline-related injuries (23%) (Figure S1, see Supplementary data). 9 patients presented with nerve injury prior to surgery (7 ulnar, 1 median, 1 posterior interosseous nerve) and all of them had no ESIN removal. 4 underwent surgical exploration. Recovery was observed in all cases, although two had persistent but improved symptoms.

**Table 1:** Baseline characteristics of the cohort. Values are number (percentage) or as specifed

Variables	ESIN retention (n = 152)	ESIN removal (n = 136)
Age, years, mean (SD)	8.9 (3.0)	9.5 (2.9)
Height, m, mean (SD)	1.39 (0.2)	1.42 (0.2)
Weight, kg, median (IQR)	32 (24–42)	37 (26–47)
Body mass index, median (IQR)	17 (15–18)	18 (16–20)
Operative time, min., median (IQR)	47 (39–67)	45 (30–61)
Sex		
Female	60 (40)	58 (43)
Male	92 (60)	78 (57)
Fracture type		
Both-bone forearm fracture	127 (84)	115 (84)
Isolated radius shaft fracture	18 (12)	16 (12)
Isolated ulnar shaft fracture	7 (4.6)	5 (3.7)
Open fracture		
No	137 (90)	125 (92)
Yes	15 (10)	11 (8.1)
Other associated injuries		
None	148 (97)	133 (98)
Present **^a^**	4 (2.6)	3 (2.2)

a Other injuries were: concussion, knee injury, metacarpal fracture, humerus fracture (n = 3), nerve injury.

Planned implant removal was recorded in 135 patients; removal was performed in 126 (93%), while 9 (7%) did not undergo removal, mainly due to patient preference. The median time to removal was 7.3 months (IQR 5.6–10.6). The mean follow-up was 6.7 (SD 2.5) years in the removal group and 2.8 (SD 1.2) years in the non-removal group. Among the 152 patients without planned routine removal, implant retention was generally well tolerated. 9 patients (6%) subsequently underwent removal due to symptoms. The main indication was implant-related irritation (n = 6), involving the wrist (n = 3), elbow (n = 2), or both (n = 1). Other indications included refracture (n = 1), infection (n = 1), and non-union (n = 1).

### Outcomes

#### Refractures

**Figure 2:**
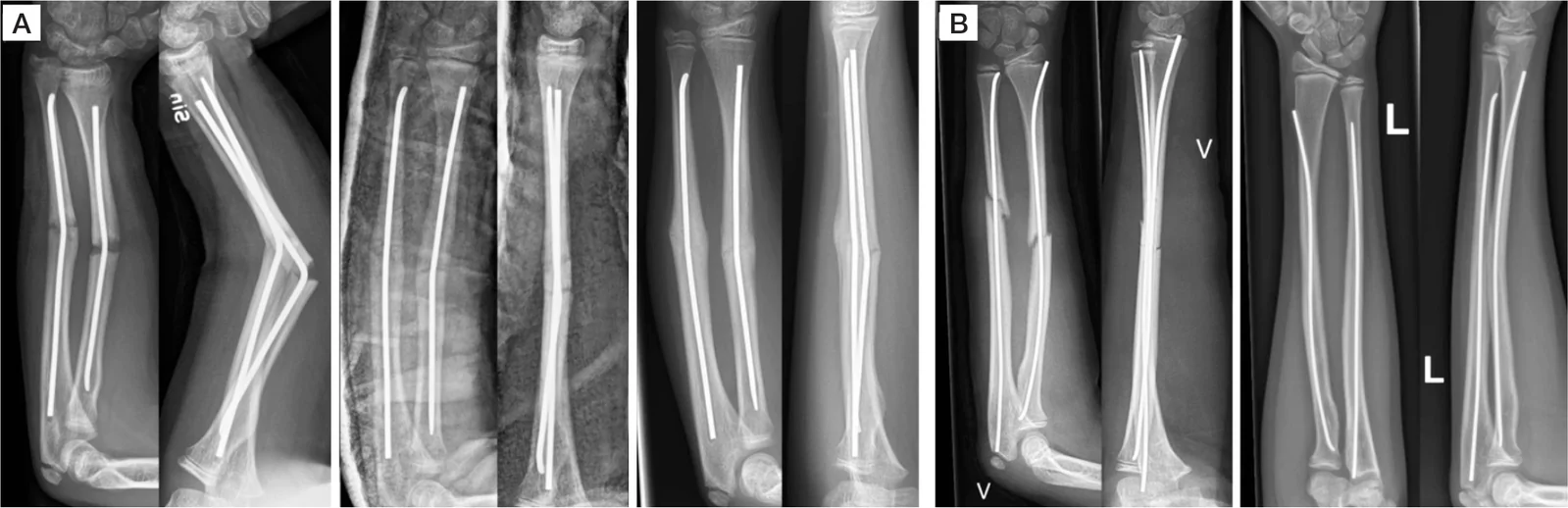
(A) Refracture with ESIN in situ 5 months after index surgery. The refracture was managed with closed reduction, with uneventful healing. The nails were not removed. (B) ESIN in situ in a 10-year-old boy. Radiographs 2 years later (right) demonstrate growth, moving the physes away from the implant. The patient remained asymptomatic, and the implants were retained.

21 refractures (7.3%) were observed during follow-up, with 4.5% occurring within the first year (Table 2). Individual case details are presented in Table S1 (see Supplementary data). Overall, refracture occurred in 13 of 136 patients (9.6%) in the removal group and in 8/152 patients (5.3%) in the non-removal group. Representative cases of refracture with ESIN in situ and continued skeletal growth with retained implants are illustrated in Figure 2.

**Table 2:** Refracture analysis and healthcare resource use according to implant removal status

Outcome	Removal (n = 136)	Retention (n = 152)	Effect estimate (CI) P value
Refracture outcomes			
Refracture overall	13 (9.6)	8 (5.3)	RD: 4.3 pp (−1.8 to 10.4);
			HR: 1.4 (0.5–3.4)
Refracture < 12 months	8 (5.9) **^a^**	5 (3.3)	RD: 2.6 pp (−2.3 to 7.5);
			OR: 1.8 (0.6–6.2)
Refracture ≥ 12 months	5 (3.7)	3 (2.0)	RD: 1.7 pp (−2.2 to 5.6);
			OR: 1.9 (0.4–9.4)
Outpatient visits after primary surgery			< 0.001 **^b^**
No visit	4 (2.9)	8 (5.3)	
1 visit	44 (32)	84 (55)	
2 visits	52 (38)	39 (26)	
≥ 3 visits	36 (26)	21 (14)	
Radiographic examinations			< 0.001 **^b^**
≤ 3	36 (26)	86 (57)	
4–5	74 (54)	56 (37)	
≥ 6	26 (19)	10 (6.6)	

a 3 refractures in the removal group occurred within 6 months following implant removal.

b Categorical variables were compared using the chi-square test.

HR = hazard ratio (Cox regression). OR = odds ratio (logistic regression). RD = risk difference. pp = percentage points.

In Cox regression analysis, refracture occurred in 13/136 patients (9.6%) in the removal group and 8/152 (5.3%) in the retention group (absolute risk difference 4.3 percentage points, CI −1.8 to 10.4; HR 1.4, CI 0.5–3.4). Most refractures occurred within the first 12 months after surgery (n = 13, 4.5%). Early refracture (< 12 months) occurred in 8/136 patients (5.9%) in the removal group and 5/152 (3.3%) in the retention group (absolute risk difference 2.6 percentage points, CI −2.3 to 7.5; OR 1.8, CI 0.6–6.2) (see Table 2).

**Figure 3:**
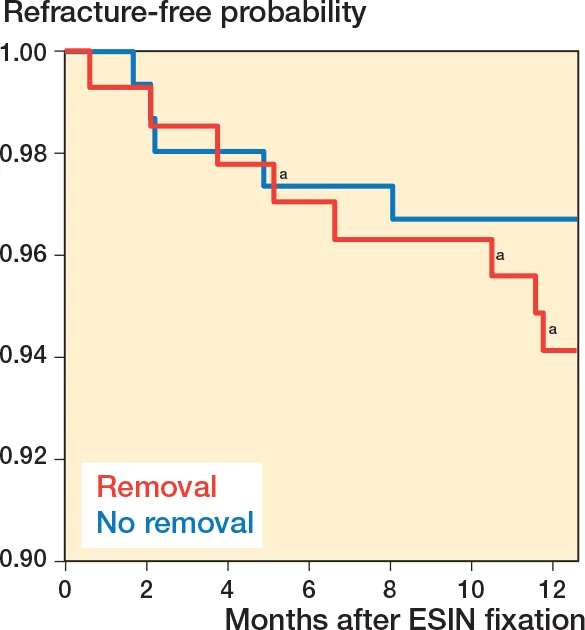
Kaplan–Meier curve of refracture-free survival after ESIN fixation according to implant removal status within the first year after surgery. ^a^ ESIN were removed when the refracture occurred.

Kaplan–Meier analysis demonstrated similar refracture-free survival between groups in the early postoperative period, with overlapping curves during the first months after surgery. A visual divergence between groups was observed later in follow-up, with more refractures occurring after implant removal, including 3 cases within 6 months following removal (Figure 3).

Refractures were commonly managed nonoperatively with casting or closed reduction and casting (see Table S1). Surgical treatment was required in selected cases, including re-osteosynthesis with ESIN or plate fixation. In a few patients, refracture occurred with implants in situ and was treated either with closed reduction with the implant in situ, or with re-nailing.

#### Follow-up resource use

Patients who underwent ESIN removal had greater follow-up resource use compared with those with retention (see Table 2). The removal group required more outpatient visits (P < 0.001) and underwent more radiographic examinations (P < 0.001). In the removal group, 26% of patients had 3 or more follow-up visits compared with 14% in the non-removal group. Similarly, 19% of patients in the removal group underwent 6 or more radiographic examinations compared with 6.6% in the non-removal group.

Overall, this corresponded to higher resource use per patient, with 4.6 vs 3.7 radiographic examinations and 2.0 vs 1.6 outpatient visits in the removal and non-removal groups, respectively. Extrapolated to the 152 patients without removal, this represents approximately 133 fewer radiographic examinations, and 68 fewer outpatient visits (reductions of about 19% and 22%) compared with expected use if resource utilization had been similar to the removal group.

#### Other complications

Following primary ESIN fixation, complications occurred in 44 patients (15%) (Table 3). Nerve injury was the most frequently observed complication (n = 14, 4.9%), predominantly affecting the radial nerve (n = 9). Most nerve injuries were transient, with complete recovery in all but 2 patients. After implant removal, complications were observed in 11 patients (8.1%). Nerve injury occurred in 3 patients (2.2%), all involving the radial nerve; all cases were transient and resolved fully. Infection occurred in 5/136 patients (3.7%) following implant removal compared with 4/288 (1.4%) following the primary ESIN procedure (RD 2.3 percentage points, CI −1.2 to 5.7; Fisher’s exact P = 0.2). Refracture within 3 months occurred in 2/136 (1.5%) following implant removal and 5/288 (1.7%) following the primary ESIN procedure.

**Table 3:** Other complications following ESIN fixation and implant removal. Values are count (%)

Complication type	After ESIN surgery (n = 288)	After ESIN removal (n = 136)
Infection	4 (1.4)	5 (3.7)
Nerve injury	14 (4.9)	3 (2.2)
Malpositioned ESIN	2 (0.7)	0
Malreduction	2 (0.7)	0
Bleeding	1 (0.3)	0
Reoperation	2 (0.7)	1 (0.7)
Other	14 (4.9)	0
Refracture within 3 months	5 (1.7)	2 (1.5)
Total complications	44 (15)	11 (8.1)

## Discussion

To our knowledge, this is one of the first studies to compare these strategies following a change in clinical practice, while simultaneously evaluating multiple outcomes, including refracture risk, complications, and healthcare resource use. We aimed to evaluate refracture risk following ESIN retention compared with implant removal and assessed complications and healthcare resource use. We found a low overall refracture rate of 4.5% observed in the first year. No clear difference in refracture risk was observed between the 2 groups. Notably, 3 early refractures occurred within 6 months after implant removal, suggesting a potential period of increased vulnerability following removal. Implant retention was associated with decreased healthcare utilization, including fewer outpatient visits and radiographic examinations.

The observed refracture rate is consistent with previous literature [14]. Notably, approximately one-third of refractures in the ESIN group occurred after prior implant removal, suggesting that removal may contribute to an increased risk of subsequent fracture. This supports the concept that ESIN provides effective stabilization during early healing, whereas disruption of this stability, such as through implant removal, may transiently increase fracture risk.

Our findings also align with recent evidence supporting implant retention. Brattgjerd et al. reported that refractures occurred in approximately 6% of patients and were primarily early events, with some occurring after implant removal, while implant retention was generally associated with few symptoms [8]. In addition, nail prominence at the entry site has been identified as an important cause of implant-related symptoms and subsequent implant removal [8]. In our cohort, symptomatic implant removal was 7%, which may be related to the practice of cutting the nail ends shorter and leaving them flush with the cortex, while maintaining the same nail entry sites and insertion technique throughout the study period. In addition, in a small cohort of pediatric femoral fractures treated with ESIN, no difference in residual pain was observed between patients who underwent implant removal and those with retained implants [15]. Together with our results, this suggests that implant retention is generally safe and well tolerated, and that routine removal may not be necessary in asymptomatic patients.

A key finding of our study is the decreased healthcare utilization associated with implant retention. Patients undergoing removal required significantly more outpatient visits and radiographic examinations. This reflects a greater demand on healthcare resources, with implications for both cost and system capacity, but also affects children and parents by increasing school and work absenteeism. In addition, repeated imaging contributes to cumulative radiation exposure in a pediatric population, which should be minimized whenever possible [16]. In our cohort, retaining implants was associated with approximately 133 fewer radiographic examinations and 68 fewer outpatient visits, as well as avoidance of 136 day-surgery procedures for implant removal, indicating a substantial reduction in healthcare utilization.

These findings should also be interpreted in the context of a broader shift toward increased surgical management of pediatric forearm fractures [17]. In this context, our results suggest that not only the initial surgical indication but also subsequent procedures, such as routine implant removal, should be critically reconsidered to avoid potential overtreatment.

An additional consideration is the difference between pediatric and adult orthopedic practice. In adults, routine implant removal is generally not recommended, and hardware is typically retained unless symptomatic [18]. In contrast, routine removal has historically been common in children, particularly following ESIN fixation [19]. This practice has largely been based on theoretical concerns, including potential effects on growth, implant migration, corrosion, and long-term tissue reactions [20]. However, available evidence has not demonstrated clinically significant adverse effects of retained flexible intramedullary implants, although long-term data remains limited, particularly in younger children with substantial remaining growth. Given that ESIN does not cross the physis and is designed to be minimally invasive, the biological rationale for routine removal remains uncertain. In the absence of robust natural history data supporting routine implant removal after fracture healing in children, the justification for this practice remains unclear.

These findings support consideration of a shift from routine to selective implant removal, reserving removal for symptomatic patients with implant-related complications.

### Limitations

First, the retrospective design introduces the potential for selection bias and unmeasured confounding. Second, implant removal was not randomized but determined by institutional policy, which may introduce temporal bias. Third, follow-up duration differed between groups, which may have influenced the detection of late refractures, although most events occurred within the first year. Furthermore, the study may be underpowered to detect moderate differences in refracture risk, as reflected by the wide confidence intervals. The low number of refracture events increases the risk of a type II error, meaning that clinically relevant differences between groups may not have been detected. Finally, analyses were limited to univariable models due to the low number of events.

### Conclusions

No clear difference in refracture risk was observed between patients undergoing ESIN removal and those retaining their ESIN implants. Implant retention was associated with lower healthcare resource use. These findings suggest that routine removal of ESIN implants in asymptomatic patients may be unnecessary. Implant removal may be reserved for patients who develop symptoms or implant-related discomfort.

### Supplementary data

Figure S1 and Table S1 are available as supplementary data on the article home page, doi: 10.2340/17453674.2026.46804

## Supplementary Material



## Notes

PHR, RBJ, RO: study design and conception. PHR, AEH, EBH: data acquisition. MT: data analysis and interpretation. MT, AEH, EBH: writing—original draft. PHR, RBJ: supervision. All authors: writing—review and editing.

The authors thank Johanna Austeen Gjestland for facilitating data handling

Handling co-editors: Ilkka Helenius and Robin Christensen

*Acta* thanks Klaus Dieter Parsch and Antti Joonas Stenroos for help with peer review of this manuscript.
