# Supplementary material for: Should we remove flexible intramedullary nails after diaphyseal forearm pediatric fracture? A cohort study of 288 patients
Source: Acta Orthop. 2026 Sep 14;97:665–71. doi: 10.2340/17453674.2026.46804 (PMC13572937; doi:10.2340/17453674.2026.46804)
Supplement: Supplementary file 1 [file ActaO-97-46804-s1.pdf]

Supplementary Material

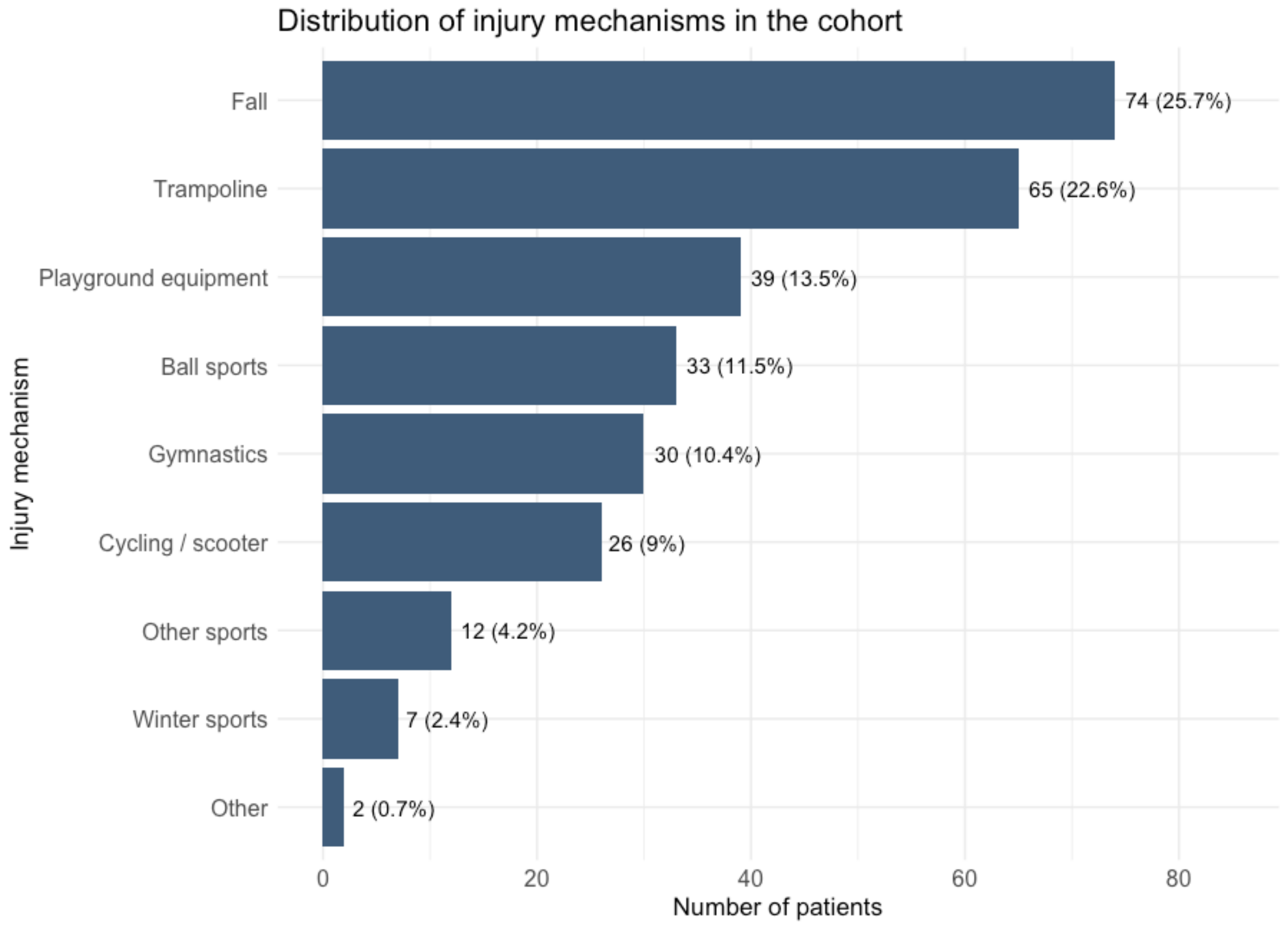

Figure S1. Distribution of injury mechanisms in the study cohort

ID	Sex	Age at injury (years)	Initial fracture type	ESIN removed	Planned removal	Refracture with ESIN in situ	Refracture <12 months	Time to refracture (months)	Time from removal to refracture (months)	Treatment of refracture
1	F	11	Isolated radial	Yes	Yes	Yes	Yes	2.1	–	New ESIN
2	F	7	Both-bone forearm	Yes	Yes	Yes	Yes	11.5	–	Casting (Fracture distal to the ESIN nails)
3	F	10	Both-bone forearm	Yes	Yes	Yes	Yes	0.6	–	Casting
4	M	12	Isolated radial	Yes	Yes	Yes	No	12.7	–	Plate fixation (Refracture also in 2022, treated with ESIN)
5	F	10	Both-bone forearm	Yes	Yes	Yes	Yes	6.3	–	Closed reduction, Nail removed later and reinserted
6	F	6	Both-bone forearm	Yes	No	Yes	Yes	3.7	–	Two refractures. Three nail removals. Possible infection after second nail removal
7	M	13	Both-bone forearm	Yes	Yes	No	Yes	5.1	0.9	New ESIN
8	M	10	Both-bone forearm	Yes	Yes	No	Yes	11.7	4.9	New ESIN
9	M	6	Both-bone forearm	Yes	Yes	No	Yes	10.4	1.3	Closed reduction/Casting
10	M	10	Both-bone forearm	Yes	Yes	No	No	40.9	30.6	Closed reduction/Casting
11	M	4	Both-bone forearm	Yes	Yes	No	No	39.7	32.1	Casting/New ESIN
12	M	9	Both-bone forearm	Yes	Yes	No	No	54.4	48.8	Casting
13	M	11	Isolated radial	Yes	Yes	No	No	56.6	49.1	Casting
14	M	7	Both-bone forearm	No	Yes	Yes	Yes	2.1	–	Closed reduction/Casting
15	M	14	Isolated radial	No	No	Yes	Yes	2	–	Closed reduction/Casting
16	M	4	Both-bone forearm	No	No	Yes	Yes	1.7	–	New ESIN inserted after 2 weeks of casting
17	M	12	Isolated radial	No	No	Yes	Yes	7.9	–	Closed reduction
18	F	10	Both-bone forearm	No	No	Yes	Yes	4.8	–	Closed reduction/Casting
19	F	6	Both-bone forearm	No	No	Yes	No	34.5	–	Casting
20	M	12	Both-bone forearm	No	No	Yes	No	28.4	–	Casting (Nail in ulna, pin in radius. Refracture in the radius. Planned nail removal due to symptoms from the nail)
21	F	6	Both-bone forearm	No	No	Yes	No	19.9	–	Casting

Table S1. Characteristics and management of patients with refracture (n = 21)
Abbreviations - F: Female, M: Male
